# Offline EEG hyper-scanning using anonymous walk embeddings in tacit coordination games

**DOI:** 10.1371/journal.pone.0288822

**Published:** 2023-07-20

**Authors:** Inon Zuckerman, Dor Mizrahi, Ilan Laufer

**Affiliations:** Department of Industrial Engineering and Management, Ariel University, Ariel, Israel; Jeonbuk National University, REPUBLIC OF KOREA

## Abstract

In this paper we present a method to examine the synchrony between brains without the need to carry out simultaneous recordings of EEG signals from two people which is the essence of hyper-scanning studies. We used anonymous random walks to spatially encode the entire graph structure without relying on data at the level of individual nodes. Anonymous random walks enabled us to encapsulate the structure of a graph regardless of the specific node labels. That is, random walks that visited different nodes in the same sequence resulted in the same anonymous walk encoding. We have analyzed the EEG data offline and matched each possible pair of players from the entire pool of players that performed a series of tacit coordination games. Specifically, we compared between two network patterns associated with each possible pair of players. By using classification performed on the spatial distance between each pair of individual brain patterns, analyzed by the random walk algorithm, we tried to predict whether each possible pair of players has managed to converge on the same solution in each tacit coordination game. Specifically, the distance between a pair of vector embeddings, each associated with one of the players, was used as input for a classification model for the purpose of predicting whether the two corresponding players have managed to achieve successful coordination. Our model reached a classification accuracy of ~85%.

## 1. Introduction

Previous electrophysiological research examining the coordination between players in the context of coordination games usually rely on studying the connectivity patterns between two brains of players that participated in a hyper-scanning recording scheme. Hyper-scanning allows gathering the information flow coming from each of the two brains simultaneously. In this kind of studies, measures related to the link between brain regions are usually being employed, such as inter-brain phase synchronies in different frequency bands and the functional connectivity patterns may be described by a directed weighted graph. These methods identify temporal dynamics in certain frequency range associated with specific brain regions [1, 2].

In this paper we present a novel method to examine the synchrony between brains without the need to carry out simultaneous recordings of EEG signals from two people which is the essence of hyper-scanning studies. That is, in our study the EEG recording was only of one player at a time, and the measure of the magnitude of synchronization between brains was computed algorithmically offline. Whereas in the previously mentioned studies each player tried to coordinate with a real partner while being engaged in a repeated game with feedback, in our study each player played against an anonymous player but no feedback was provided. This is a major difference, since in the previous studies the feedback affected the strategy of the player and consequently the resulting brain inter-synchronization patterns.

Moreover, another major difference is that our suggested method is oblivious to the identity of either the electrodes or the brain regions involved in the different EEG patterns associated with the two players. Specifically, in the current study we used anonymous random walks to spatially encode the entire graph structure without relying on data at the level of individual nodes. Anonymous random walks enable us to encapsulate the structure of a graph regardless of the specific node labels. That is, random walks that visited different nodes in the same sequence will result in the same anonymous walk encoding [3]. Random walk is a relatively recent technique that has been employed in various studies in conjunction with other methods such as Bayesian reasoning [4] and microstate analysis [5] to study the EEG patterns for different purposes. For example, in the former study a search for the most salient encoded pathways was carried out. In our study, however, we used lower dimensional representation of the graph and thus preserved the graph’s topology. Consequently, the classification [6] that we performed using standard methods, was employed on a feature vector that reliably encapsulates the spatial features of each network configuration.

In this study we have analyzed the EEG data offline and matched each possible pair of players from the entire pool of players that performed a series of tacit coordination games [7, 8]. Specifically, we compared between two network patterns associated with each possible pair of players. By using classification, that was performed on the spatial distance between each pair of individual brain patterns, analyzed by the random walk algorithm, we tried to predict whether each pair of players has managed to converge on the same solution in each tacit coordination game. The vector embeddings were used as input for a classification model to predict whether the two players have managed to achieve successful coordination. Our model reached a classification accuracy of ~85%.

## 2. Materials and methods

In this study participants (n = 10, mean age = ~26, SD = 4) performed a tacit coordination task while EEG was recorded from their scalp. The task required to select a single word from a list of four words presented horizontally on the computer screen. Noteworthily, each participant sat alone in front of the computer screen and had to select a word as if she was coordinating with an unknown partner. Participants were given a monetary incentive for a successful coordination and were told that their performance will be evaluated, that is, that their responses will be randomly paired with another participant from the pool of participants.

**Fig 1:**
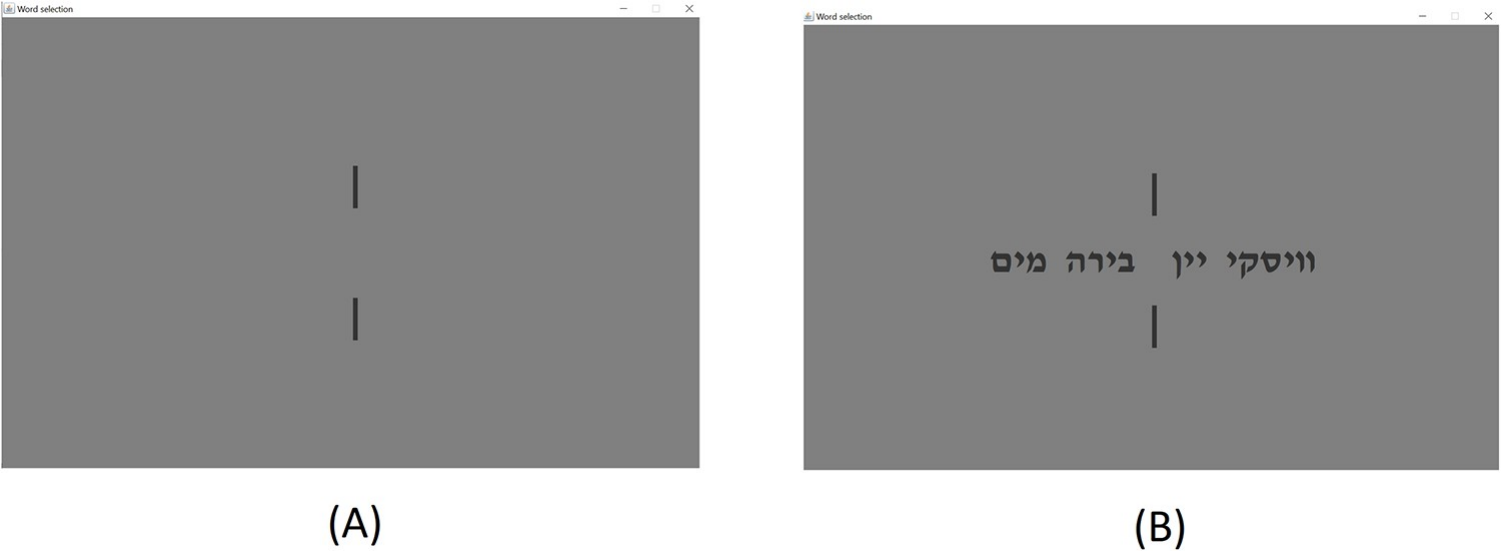
(A) Stand by screen (B) Game board #1 ["Water", "Beer", "Wine", "Whisky"].

Each experimental session consisted of twelve tacit coordination games, each with a different set of four words (see Appendix A in [9]). For example, game board #1 displays the set {"Water", "Beer", "Wine", "Whisky"} appearing in Hebrew. Each set of words was displayed between two short vertical lines following a slide containing only the lines without the words, so that participants will focus their gaze at the center of the screen. Fig 1A presents a stand-by slide while Fig 1B displays the coordination task slide. Each coordination task slide was presented for a maximal duration of 8] sec] and was preceded by the stand-by slide presented for *U(2*,*2*.*5*) sec. The sequence of the task trials was randomized in each session. Before performing the actual experiment, each participant underwent a training session comprising a total of five trials (each including a different set of words) to get them familiar with the application and the EEG cap.

The EEG was recorded by 16 active channels using the international 10–20 system. We used a g.USBAMP biosignal amplifier (g.tec, Austria) at a sampling frequency of 512Hz and OpenVibe [10] as the recording software. Impedance of all electrodes was kept below the threshold of 5K [ohm].

### 2.1 Ethical statement

The Experimental protocols used in this work were evaluated and approved by the Ethic Committee of Ariel university (confirmation number: AU-SOC-SL-20190901). Permission to perform the electrophysiological recordings in the experiment was given from September 1, 2019 to August 31, 2020. Written informed consent was obtained from all subjects involved in the study.

## 3. Data processing & analysis

### 3.1 Pre-processing pipeline, data labelling and graph construction

The EEG data underwent the following preprocessing pipeline as was previously used in [9, 11–13]: band-pass filtering (BPF) [1], artifact removal following iCA, re-referencing to the average reference, baseline correction, and down sampling from 512 Hz to 64 Hz. Data was analyzed on a 1-second epoch window from the onset of each task.

Next, coherence (ranged [0,1]) was calculated for each pair of electrodes on each of the 1-sec epochs. Coherence is a statistical measure that is used to examine the relationship between two acquired signals. The EEG coherence measures the level of synchronization between two brain regions of the same person or alternatively the compatibility in brain activity of the same area between two different people [14, 15]. In our study it was defined that when the absolute coherence value was ≥0.5 it was considered that synchronization existed between that pair of electrodes, otherwise, there was no synchronization.

**Fig 2:**
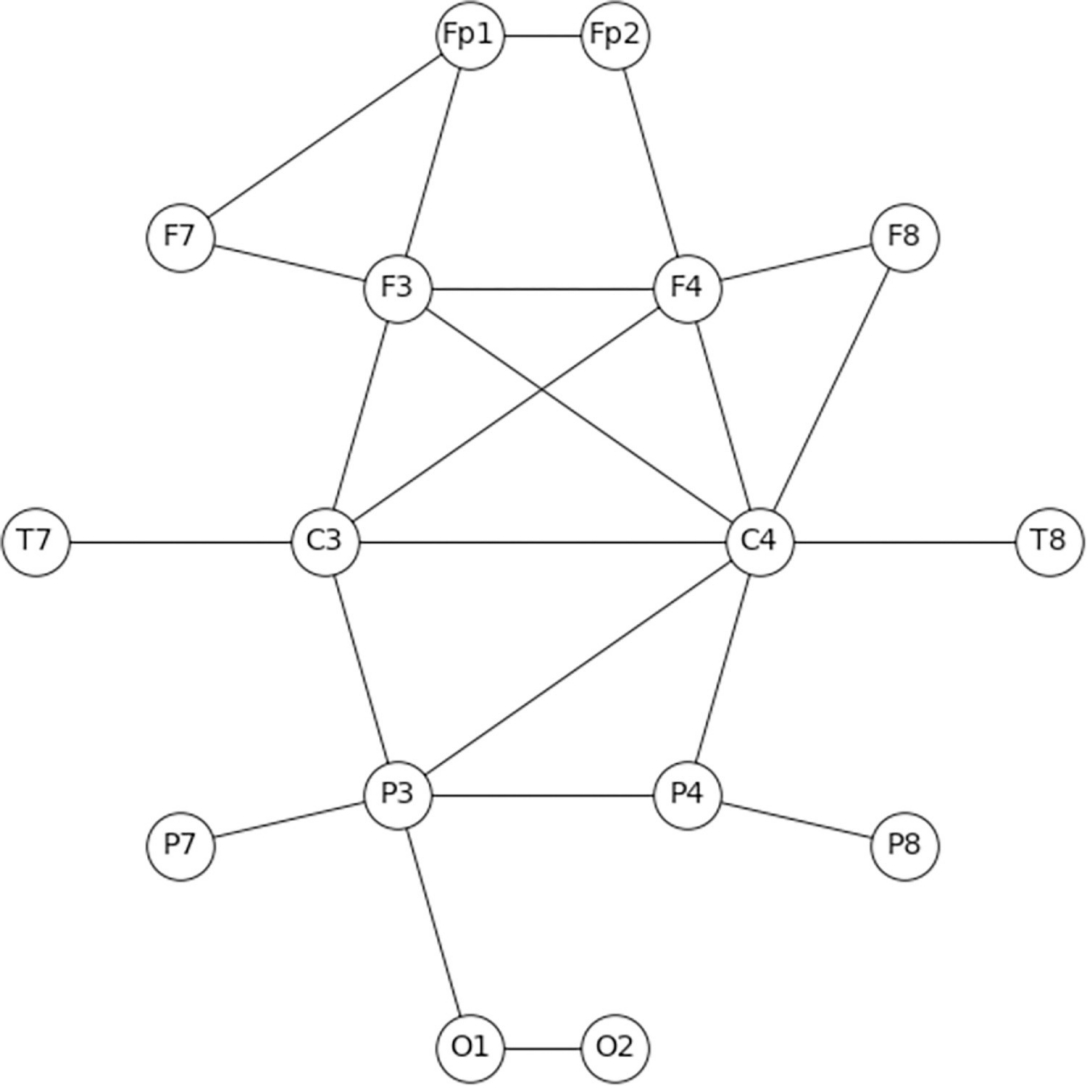
Coherence graph representation. Graph layout is in accordance with electrode placement on the scalp.

A graph comprised all viable connections between pair of electrodes. Fig 2 presents the graph obtained for an exemplar player in a specific coordination task. For example, since Fp1 has viable connections with Fp2, F3 and F7 the nodes representing these electrodes are connected to the Fp1 node. The resulting graph is undirected and its adjacency matrix (16*16) is symmetric. The maximal number of total edges in each graph (i.e., in each epoch) is therefore 120 ($\frac{n*(n-1)}{2}$), where n is the number of nodes. Each electrode in the graph (e.g., each node) can have a maximum of 15 edges excluding self-loops, which were excluded, since a coherence of a signal with itself is always one.

### 3.2 Classification of coherence graph pairs based on anonymous walks embeddings

First, before performing the classification process, we would like to complete a stage of graph embedding (e.g. [9, 16–18]). Graph embedding is a process that defines a constant length vector that represents a graph entity, i.e., a node, a sub-graph, or an entire graph. These vectors, called embeddings, are a lower dimensional representation of the graph (i.e., in relation to the adjacency matrix) and preserve the graph’s topology. This process will allow us to perform classification using standard methods of classification on a feature vector that reliably represents the features of the graph.

To be able to produce an optimal classification process, we would like to represent the graph in a way that will preserve its spatial properties, which have been proven to be critical for classifying brain processes associated with the spatial structure of the EEG data [9, 19, 20]. The random walk embedding method assumes that collecting all the single random walks into a probability density function, out of all possible walks, will describe the spatial structure of the graph regardless of the specific node labels where the walks were performed. That is, random walks that visited different nodes in the same sequence will result in the same anonymous walk encoding [3].

**Fig 3:**
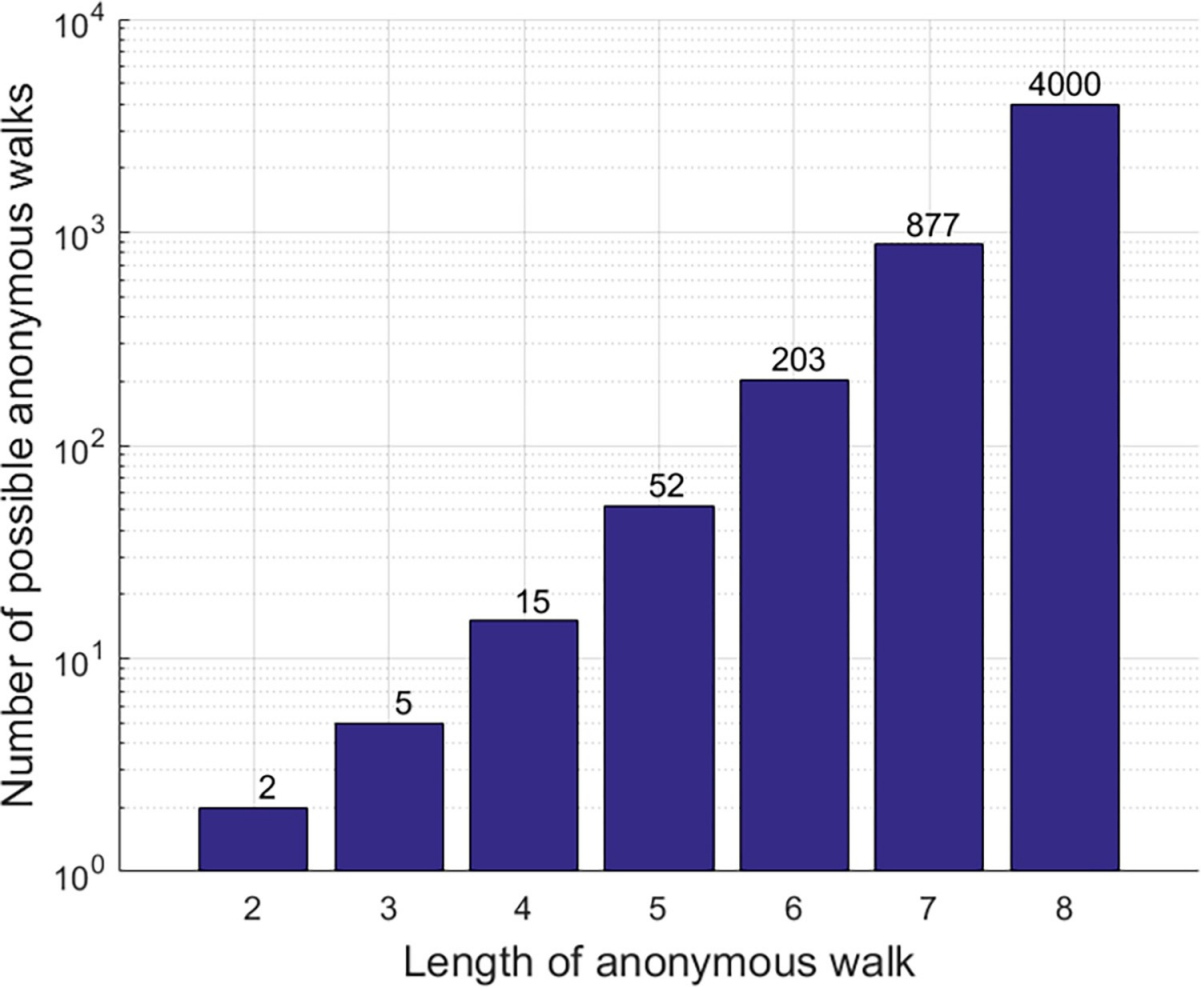
Number of possible anonymous walks in relation to walk length.

One of the main hyperparameters in the random walk graph embedding method is the random walk length. The walk length directly affects the number of possible walks which defines the size of the embedding vector. A random walk that is too short will not succeed in producing long enough path patterns that will preserve the spatial structure of the graph, while too long walks will produce patterns that are too complex that may result in overfitting. Fig 3 illustrates the exponential growth of the number of possible walks, which defines the size of the embeddings as a function of the length of the random walk. For each random walk of length n, we can construct an embedding vector whose number of elements is equal to the number of possible walks, where each element represents the probability value of performing this specific walk out of all possible walks.

To construct the embedding which contains the probability density values for the different walks there is a need to perform the appropriate number of random walks to converge to the expected probability values comprised in the embedding. As the length of the random walk increases, there is a need to perform a greater number of walks to produce an embedding vector that faithfully represents the graph. The minimal number of the random walks needed to achieve a reliable distribution was defined in [21] and the definition is presented in Eq 1:

$$m=\frac{2}{\varepsilon^{2}}(\log\left(2^{N}-2\right)-\log\left(\delta \right))$$ (Eq 1)

Where N (the Y-axis in Fig 3) is the number of possible anonymous walk patterns for an anonymous walk of a specific length (the X-axis in Fig 3). The distribution of anonymous walks has an error of no more than (ε) with probability less than (δ) relative to the optimal distribution (*m*→∞). In this study we determined the number of random walks required (m) using Eq 1 by setting ε = 0.1 and δ = 0.01.

After we have performed the embedding process, we will want to perform a classification for each pair of players and the corresponding embedding vectors and predict whether the players were able to coordinate their answers. To construct the data set for supervised learning, we have first labeled each pair of players in each coordination game. Specifically, if both players chose the same solution in the coordination game, we labeled the pair of graphs as positive (i.e., "1"); if both players chose a different solution, we labeled the pair of graphs as negative (i.e., "0").

Our feature vector for the classification model will be derived by calculating the difference vector, z (see Eq 2), which is the absolute difference of the two embedding vectors (e_1_ and e_2_), each belonging to one of the players from the pair which are compared with each other:

$$z=|e_{1}-e_{2}|$$ (Eq 2)

**Fig 4:**
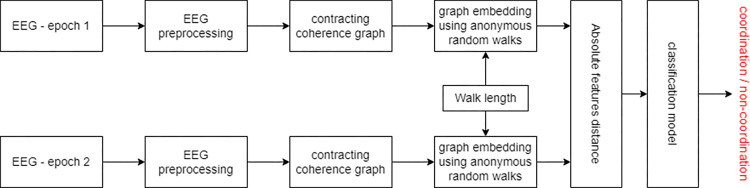
Classification pipeline.

Next, the set of the difference vectors alongside their appropriate label served as the input to the XGBoost classifier [22] to train the model. To get optimal classification results, we optimized the following hyperparameters of the model using grid-search: number of estimators, maximal depth, and learning rate. The entire classification pipeline is displayed in Fig 4.

**Fig 5:**
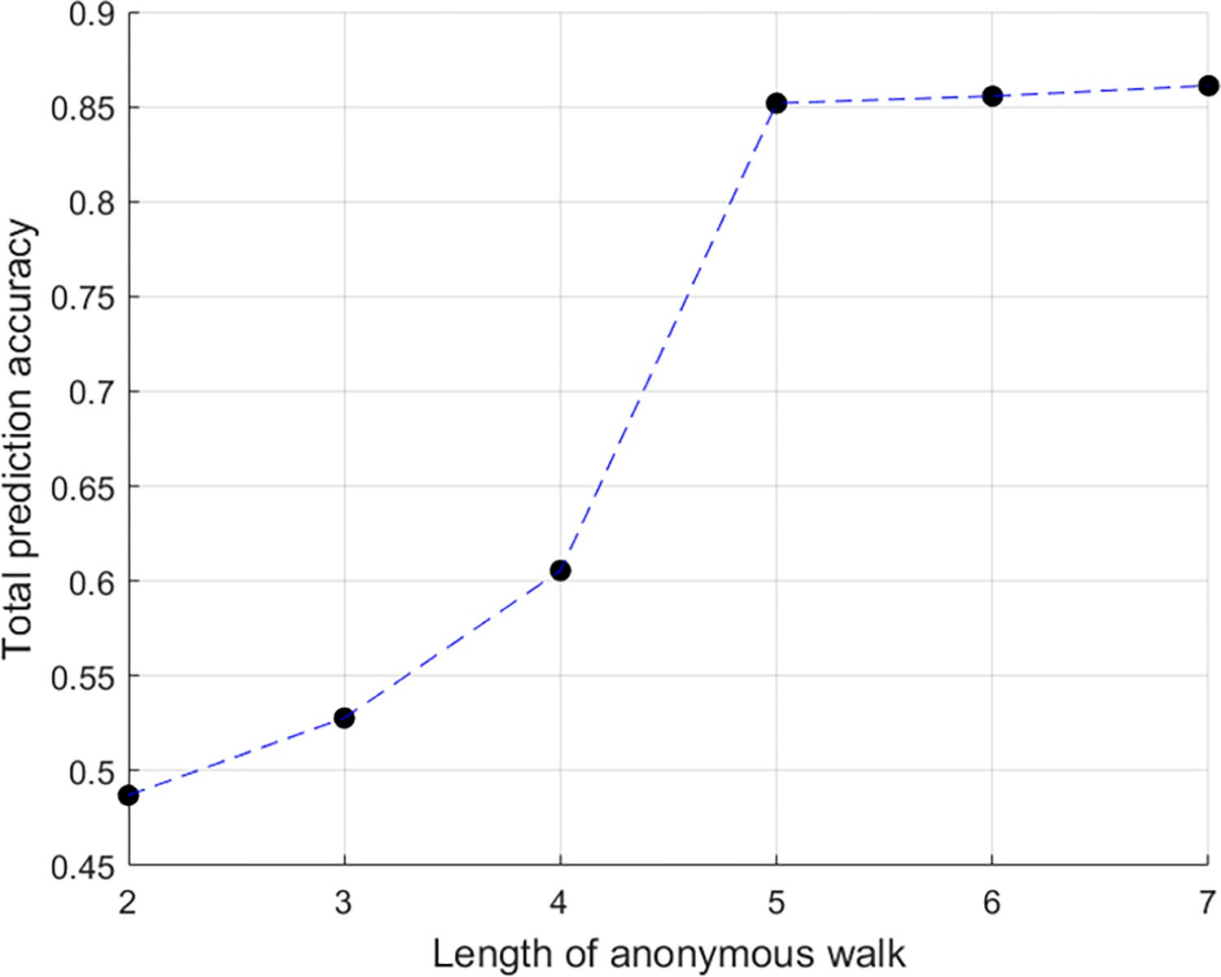
Classification accuracy as a function of random walk length.

A comparison of the ten players with each other in the twelve coordination tasks produced a dataset that included 540 observations divided between the two classes, 153 positive observations (i.e., coordination) and 387 negative observations (i.e., lack of coordination). To avoid over-fitting, we worked with a three-fold cross validation method, and each fold was equally distributed between classes (51 positive observations and 129 negative observations). For each random walk length, we have trained a specific classifier optimized by the various XGBoost hyperparameters (number of estimators, maximal depth, and learning rate). Fig 5 shows that the optimal random walk length is of size 5 since we got significant improvement in the classification results compared to previous walks (85.19% compared to 60.56%). Increasing the length of the walk beyond 5 does not yield a significant improvement (0.37% when increasing from 5 to 6, 0.56% when increasing from 6 to 7) and therefore the walk length of 5 was determined as the optimal value.

Table 1 details the results of the classification process. Since the data set is unbalanced (387 negative instances, and 153 positive instances) we report the precision ($\frac{TP}{TP+FP}$) and recall ($\frac{TP}{TP+FN}$) indices of each class separately rather than the overall accuracy percentage. For the non-coordination class, the classifier is able to classify the EEG segments with a high level of accuracy given by the positive predicted value (PPV) and true positive rate (TPR) which were each over 87% (91.15% and 87.86%, respectively). The classification indices for the coordination EEG segments, i.e., the PPV and the TPR, are a bit lower and are 71.86% and 78.43%, respectively.

**Table 1:** Classification results using anonymous walk of length 5.

		Predicted Classes		True Positive Rate	False Negative Rate
		“0” Non-coordination	“1” Coordination		
**True Classes**	“0” Non-coordination	340	47	87.86%	12.14%
	“1” Coordination	33	120	78.43%	21.57%
Positive Predicted Value		91.15%	71.86%	**Prediction Accuracy (460/540) 85.19%**	
False Discovery Rate		8.85%	28.14%		

when examining human behavior, there is no single answer that is a "correct" one. A tacit coordination game can have multiple answers from different humans and they are all correct [8]. When trying to predict what most humans would do, it can be seen from previous work that 85% accuracy is a good and acceptable result (e.g. [23–25]).

In this study we have compared between three different classification algorithms families: 1. classical machine learning techniques (e.g., multivariate logistic regression and SVM), 2. Neural network-based techniques, 3. tree-based algorithms (e.g., random forest and XGBoost). Of all the other methods that we have examined, the results consistently indicated that XGBoost provided the best overall performance, thus justifying its selection as the best method for our study.

The classification results achieved were compared to a baseline embedding method, namely the Bag-of-Node-Degree, which projects the graph onto a lower space and generate a new feature vector. The Bag-of-Node-Degree embedding method is much simpler and computationally efficient method [26] in relation to the anonymous random walk embedding method. This method measures the degree of each node spirally on the graph and generates an equivalent probability density function in relation to anonymous random walk which encapsulate the spatial properties of the graph. In order to be able to compare the models we have used the same classification model, i.e., XGBoost. The corresponding classification results of the Bag-of-Node-Degree embedding combined with XGBoost generated accuracy of 72%.

## 4. Conclusions & future work

During EEG recordings the brain electrical activity is projected onto the scalp and the brain potentials produce a spatial pattern captured by an array of scalp electrodes. In this study we propose a method to utilize the resultant discrete representation of potential topography to assess the degree of coordination between players in the context of tacit coordination games. To that end, in this study we have presented a novel method for inter-brain hyper-scanning analysis without the need to perform concurrent recordings of brain activity from two participants. The crux of the method is that individual EEG data sets are analyzed separately offline and the similarity between the participants’ electrode network configuration is computed using embedding vectors created by random walks. Our suggested offline hyper-scanning method was used to predict whether players in a tacit coordination game have managed to successfully converge on the same solution, i.e., achieve a successful coordination. While previous studies relied on temporal dynamics in various frequency ranges associated with different brain regions when trying to correlate between inter-brain networks, e.g., [1, 2, 27], our method relies only on the similarity in the networks’ spatial configuration without the need to consider specific electrode positions or frequency bands. Following three-fold cross validation training on an ensembled decision tree model (XGBoost), we have managed to achieve a classification accuracy of ~85%.

Since the data set is unbalanced (387 negative instances, and 153 positive instances) we must consider the precision and recall indices of each class. The classification results show that the PPV and TPR indices of the coordination class are lower by ~19.3% and ~ 9.4%, respectively, than their corresponding values in the coordination class (see Table 1). These differences might be explained by the difficulty level of the coordination problem at hand. Specifically, if the coordination problem is relatively easy, there is a more prominent solution the players can converge on, and consequently the spatial configuration of the brain patterns might be more similar to each other. On the other hand, when the coordination problem is relatively difficult, a prominent solution is not clear, or there might be several prominent solutions, and so the variability in the induced brain patterns is enhanced leading to an overall lower similarity in the spatial configuration of the compared brain patterns. Thus, the difficulty level of the coordination problems presented in each of the tacit coordination games, might affect both precision and recall, which are lower in the case of the coordination class.

There are some limitations of the current study that warrant consideration. First, in this study, all the participants were students and belonged to the same university. Thus, in future work it is necessary to include diverse populations in the pool of participants to even-out the effect of cultural background [28] as much as possible. Second, the aim of the current study was to predict successful coordination by using an offline simple technique of hyper-scanning. It would be worthwhile to examine the effectiveness of the proposed method in other contexts such as social dilemmas (Prisoner’s dilemma, Ultimatum Game), coordination with diverge interest (e.g. [29]), and repeated interactions (repeated Prisoner’s dilemma). We expect that the similarity between network configurations will increase as the game progresses. Noteworthily, in our task there was no feedback following the player’s decision in each trial as in the case of repeated interactions. Third, in our study we have used the similarity measure to predict successful coordination. However, this measure could also be used to examine the effect of other variables such as the degree of coordination, type of cues (e.g., verbal vs. nonverbal), as well as behavioral traits such as social value orientation [29] and loss aversion [30, 31]. Lastly, the fourth limitation pertains to the number of electrodes we used for EEG recording. The electrode cap included only 16-channels which reduced computational complexity. That is, increasing the number of electrodes (e.g., to 32 or 64) would exponentially increase the average length of the anonymous walk and will therefore entail increasing the number of participants as well and the algorithm’s running time. On the other hand, it is worth considering using a higher number of electrodes, which will potentially lead to a higher resolution of the coherence graph.

## Supporting information

S1 File (DOCX)
