# Supplementary material for: Offline EEG hyper-scanning using anonymous walk embeddings in tacit coordination games
Source: PLoS One. 2023 Jul 20;18(7):e0288822. doi: 10.1371/journal.pone.0288822 (PMC10358924; doi:10.1371/journal.pone.0288822)
Supplement: S1 File — (DOCX) [file pone.0288822.s001.docx]

S1. Tacit coordination game list results

The full list of results of all participants in the 12 different coordination games is presented in Table A. 1. Based on these results we have defined the labels that were used for the classification process of the electrophysiological segments.

Table S. 1: coordination results

| **Game number** | **Player 1** | **Player 2** | **Player 3** | **Player 4** | **Player 5** | **Player 6** | **Player 7** | **Player 8** | **Player 9** | **Player 10** |
| --- | --- | --- | --- | --- | --- | --- | --- | --- | --- | --- |
| **1** | 1 | 2 | 2 | 2 | 4 | 4 | 2 | 1 | 2 | 1 |
| **2** | 4 | 2 | 4 | 2 | 4 | 1 | 4 | 3 | 4 | 3 |
| **3** | 3 | 1 | 1 | 3 | 3 | 1 | 3 | 2 | 1 | 4 |
| **4** | 4 | 3 | 1 | 1 | 3 | 2 | 2 | 4 | 1 | 4 |
| **5** | 4 | 4 | 4 | 4 | 2 | 4 | 2 | 2 | 2 | 3 |
| **6** | 2 | 3 | 1 | 1 | 4 | 2 | 2 | 2 | 1 | 4 |
| **7** | 3 | 3 | 2 | 2 | 2 | 3 | 3 | 2 | 2 | 3 |
| **8** | 4 | 3 | 3 | 4 | 4 | 2 | 3 | 4 | 4 | 1 |
| **9** | 2 | 2 | 4 | 2 | 3 | 1 | 3 | 4 | 1 | 1 |
| **10** | 3 | 3 | 3 | 3 | 2 | 3 | 2 | 1 | 2 | 4 |
| **11** | 1 | 1 | 1 | 1 | 2 | 4 | 3 | 3 | 4 | 2 |
| **12** | 2 | 3 | 2 | 3 | 2 | 2 | 2 | 3 | 2 | 4 |
